# 5-Bromo­benzene-1,3-dicarbo­nitrile

**DOI:** 10.1107/S1600536813028857

**Published:** 2013-11-06

**Authors:** Nadine Seidel, Wilhelm Seichter, Edwin Weber

**Affiliations:** a Institut für Organische Chemie, TU Bergakademie Freiberg, Leipziger Strasse 29, D-09596 Freiberg/Sachsen, Germany

## Abstract

The asymmetric unit of the title compound, C_8_H_3_BrN_2_, consists of two mol­ecules. The crystal structure features undulating mol­ecular sheets with the mol­ecules linked by C—H⋯N hydrogen bonds with one N atom acting as a bifurcated acceptor. N⋯Br inter­actions also occur [N⋯Br = 2.991 (3) and 3.099 (3) Å]. Inter­layer association is accomplished by offset face-to-face arene inter­actions [centroid–centroid distance = 3.768 (4) Å].

## Related literature

For use of aromatic nitrils in organic synthesis and for their industrial applications, see: Fabiani (1999 ▶); Ishii *et al.* (2011 ▶); Sandier & Karo (1983 ▶). For uses of aromatic nitrils in crystal engineering and the construction of metal-organic frameworks, see: Desiraju & Harlow (1989 ▶); Leonard & MacGillivray (2010 ▶); Reddy *et al.* (1993 ▶); Tiekink *et al.* (2010 ▶). For the X-ray structure of 1,3,5-tri­cyano­benzene, see: Reddy *et al.* (1995 ▶). For non-covalant C—H⋯N and N⋯Br inter­actions as well as arene⋯arene stacking contacts, see: Desiraju & Steiner (1999 ▶); Dance (2004 ▶); Rowland & Taylor (1996 ▶); Steiner (2002 ▶). For the preparation of the title compound, see: Doyle & Haseltine (1994 ▶).
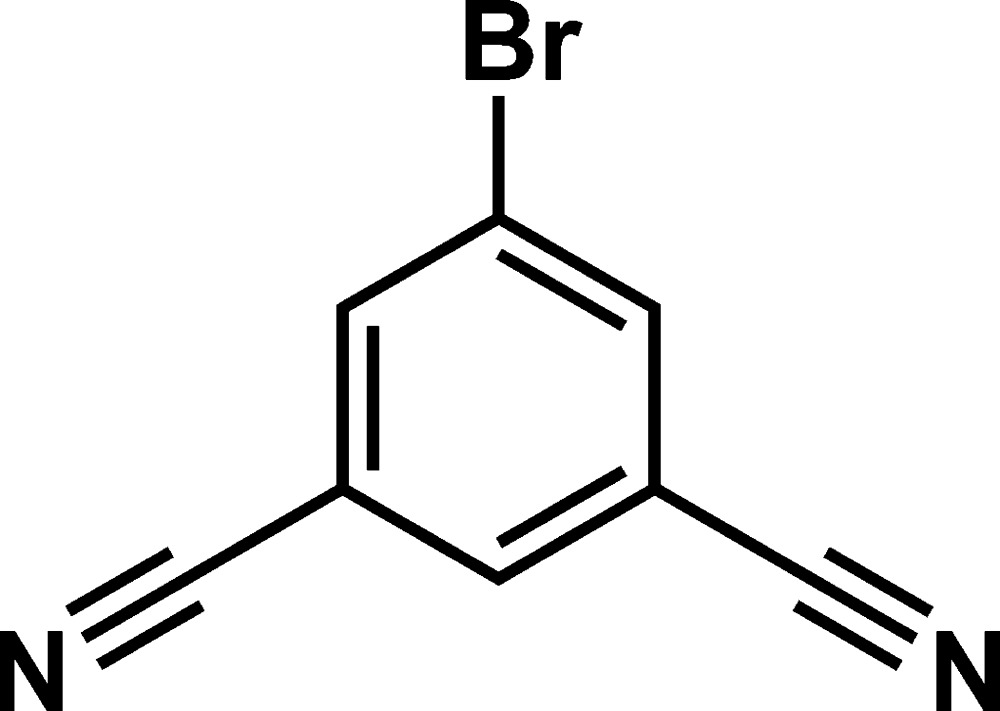


## Experimental

### 

#### Crystal data

C_8_H_3_BrN_2_
*M* *_r_* = 207.03
Monoclinic, 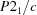

*a* = 13.3019 (4) Å
*b* = 15.7762 (5) Å
*c* = 7.4265 (2) Å
β = 93.719 (2)°
*V* = 1555.19 (8) Å^3^
*Z* = 8
Mo *K*α radiation
μ = 5.21 mm^−1^
*T* = 173 K
0.45 × 0.43 × 0.08 mm

#### Data collection

Bruker APEXII CCD area-detector diffractometer
Absorption correction: multi-scan (*SADABS*; Bruker, 2007 ▶) *T*_min_ = 0.203, *T*_max_ = 0.681
16811 measured reflections
4198 independent reflections
3436 reflections with *I* > 2σ(*I*)
*R*_int_ = 0.039

#### Refinement

*R*[*F* ^2^ > 2σ(*F* ^2^)] = 0.031
*wR*(*F* ^2^) = 0.078
*S* = 1.05
4198 reflections
199 parameters
H-atom parameters constrained
Δρ_max_ = 0.60 e Å^−3^
Δρ_min_ = −0.58 e Å^−3^

### 

Data collection: *APEX2* (Bruker, 2007 ▶); cell refinement: *SAINT-NT* (Bruker, 2007 ▶); data reduction: *SAINT-NT*; program(s) used to solve structure: *SHELXS97* (Sheldrick, 2008 ▶); program(s) used to refine structure: *SHELXL97* (Sheldrick, 2008 ▶); molecular graphics: *ORTEP-3 for Windows* (Farrugia, 2012 ▶); software used to prepare material for publication: *SHELXTL* (Sheldrick, 2008 ▶).

## Supplementary Material

Crystal structure: contains datablock(s) I, New_Global_Publ_Block. DOI: 10.1107/S1600536813028857/zp2009sup1.cif

Structure factors: contains datablock(s) I. DOI: 10.1107/S1600536813028857/zp2009Isup2.hkl

Supplementary material file. DOI: 10.1107/S1600536813028857/zp2009Isup3.cml

Additional supplementary materials:  crystallographic information; 3D view; checkCIF report

## Figures and Tables

**Table 1:** Hydrogen-bond geometry (Å, °)

*D*—H⋯*A*	*D*—H	H⋯*A*	*D*⋯*A*	*D*—H⋯*A*
C4*A*—H4*A*⋯N2^i^	0.95	2.69	3.388 (3)	130
C4—H4⋯N2*A* ^i^	0.95	2.61	3.444 (3)	147
C6*A*—H6*A*⋯N1*A* ^ii^	0.95	2.67	3.563 (3)	157
C2—H2⋯N1^iii^	0.95	2.69	3.624 (3)	168
C2*A*—H2*A*⋯N1^iii^	0.95	2.72	3.435 (3)	133

Symmetry codes: (i) 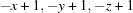; (ii) 
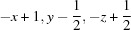
; (iii) 
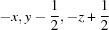
.

**Fig. 1.**
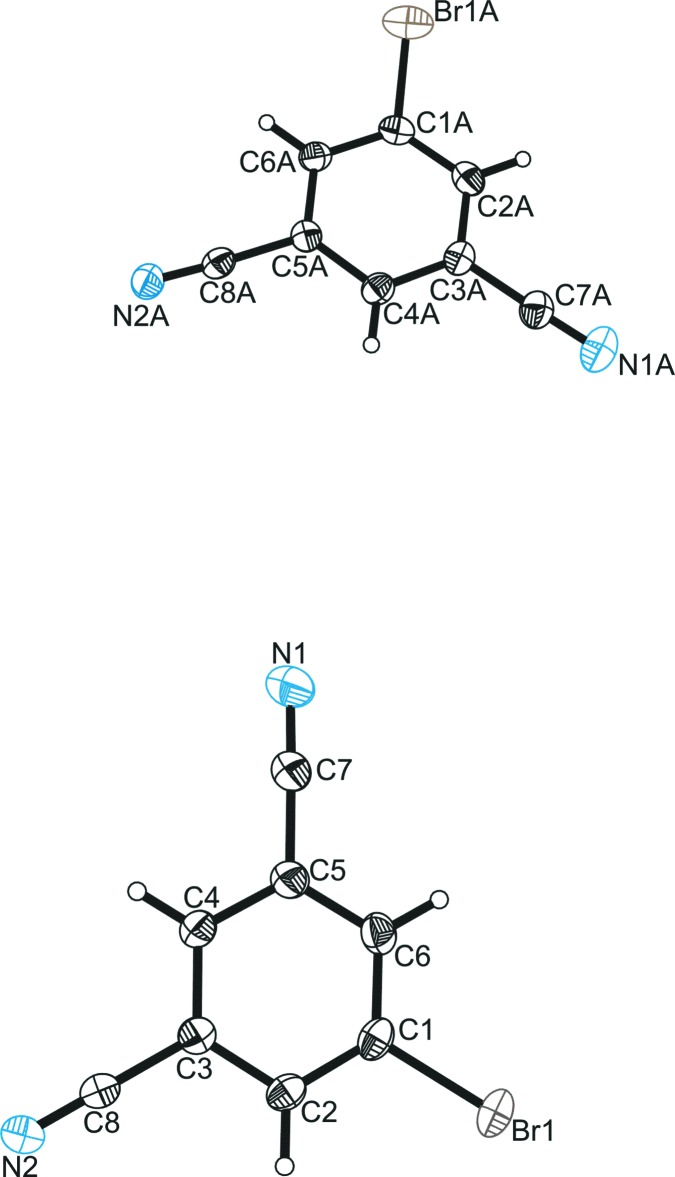
Asymmetric unit of the title compound, showing the atom numbering scheme. Displacement ellipsoids for the non-H atoms are drawn at the 50% probability level.

**Fig. 2.**
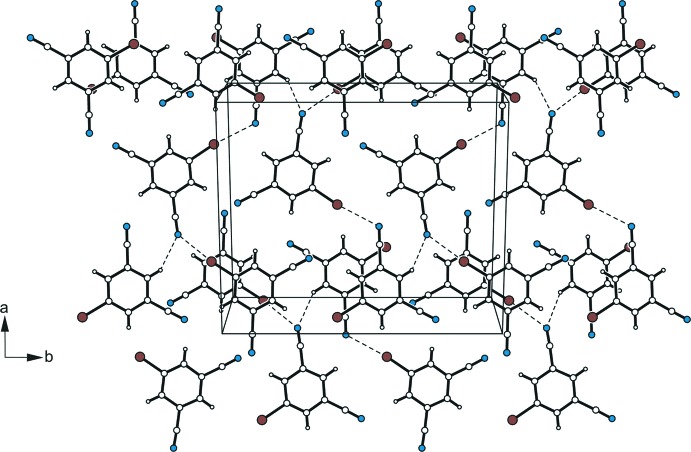
Packing structure viewed along the c-axis. Relevant intermolecular interactions are indicated as broken lines.

**Fig. 3.**
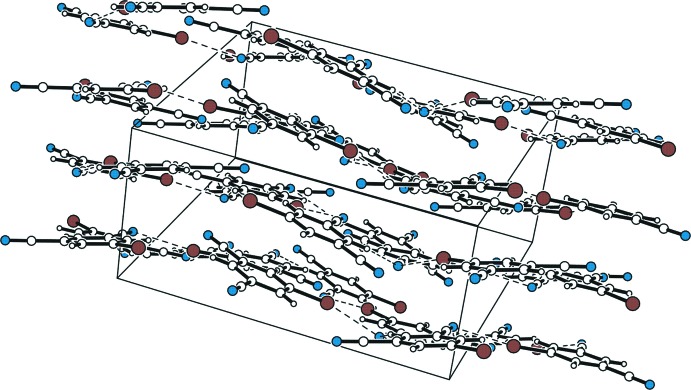
A view along the b-axis showing the intermolecular contacts as broken lines.

## supplementary crystallographic information

### 1. Comment

Aromatic nitriles are important intermediate compounds in organic synthesis (Ishii *et al.*, 2011). They can smoothly be converted into a great many of other functional groups, such as carboxylic acids, amidines, amines, esters and ketones (Sandier *et al.*, 1983). Futhermore, they are used as functional materials, pharmaceuticals, dyes and liquid crystals (Fabiani *et al.*, 1999). Recently, aromatic nitriles have also arisen interest for their capability of forming supramolecular interactions that turned out to good account in organic crystal engineering (Desiraju & Harlow, 1989; Reddy *et al.*, 1993; Tiekink *et al.*, 2010) or the construction of metal-organic framework structures (Leonard & MacGillivray, 2010). Relating to this latter topics, the title compound has been synthesized as a precursor and was identified by single-crystal X-ray diffraction. The compound crystallizes in the monoclinic space group *P*2_1_/*c* with two molecules in the asymmetric part of the unit cell (Fig. 1). The bond distances and angles within the aromatic rings agree well with those found in the crystal structure of 1,3,5-tricyanobenzene (Reddy *et al.*, 1995). According to a tilt angle of 12.3 (1) ° between the independent molecules, the crystal structure is composed of undulated molecular layers with the molecules linked by C—H···N hydrogen bonds (Desiraju & Steiner, 1999) [*d*(H)···N) 2.61 - 2.72 Å; C—H···N 114 - 168 °]. In this coordination structure (Figs. 2 and 3), the nitrogen N1 acts as a bifurcated acceptor (Steiner, 2002). Moreover, the interatomic distances between N2 and the bromo substituents of neighbouring molecules [2.991 (2) and 3.099 (2) Å], being considerably shorter than the sum of van der Waals radii of the respective atoms (3.40 Å), indicate the presence of N···Br interactions (Rowland & Taylor, 1996). In direction of the stacking axis of the molecular sheets, the crystal is stabilized by *offset face-to-face* arene interactions [*Cg_A_*···*Cg_A_* = 3.768 (4) Å] (Dance, 2004).

### 2. Experimental

The title compound was synthesized from 5-bromo-1,3-benzenedicarboxylic acid following the literature procedure (Doyle & Haseltine, 1994). Single crystals of X-ray diffraction quality were obtained as colourless plates *via* crystallization from acetone.

### 3. Refinement

H atoms were positioned geometrically and allowed to ride on their respective parent atoms, with C—H = 0.95 Å and *U*_iso_(H) = 1.2 *U*_eq_(C) for aryl.

### Crystal data

**Table:**

C_8_H_3_BrN_2_	*F*(000) = 800
*M**_r_* = 207.03	*D*_x_ = 1.768 Mg m^−^^3^
Monoclinic, *P*2_1_/*c*	Mo *K*α radiation, λ = 0.71073 Å
Hall symbol: -P 2ybc	Cell parameters from 7273 reflections
*a* = 13.3019 (4) Å	θ = 2.6–29.1°
*b* = 15.7762 (5) Å	µ = 5.21 mm^−^^1^
*c* = 7.4265 (2) Å	*T* = 173 K
β = 93.719 (2)°	Plate, colourless
*V* = 1555.19 (8) Å^3^	0.45 × 0.43 × 0.08 mm
*Z* = 8	

### Data collection

**Table:**

Bruker APEXII CCD area-detector diffractometer	4198 independent reflections
Radiation source: fine-focus sealed tube	3436 reflections with *I* > 2σ(*I*)
Graphite monochromator	*R*_int_ = 0.039
phi and ω scans	θ_max_ = 29.2°, θ_min_ = 1.5°
Absorption correction: multi-scan (*SADABS*; Bruker, 2007)	*h* = −18→18
*T*_min_ = 0.203, *T*_max_ = 0.681	*k* = −21→21
16811 measured reflections	*l* = −8→10

### Refinement

**Table:**

Refinement on *F*^2^	Primary atom site location: structure-invariant direct methods
Least-squares matrix: full	Secondary atom site location: difference Fourier map
*R*[*F*^2^ > 2σ(*F*^2^)] = 0.031	Hydrogen site location: inferred from neighbouring sites
*wR*(*F*^2^) = 0.078	H-atom parameters constrained
*S* = 1.05	*w* = 1/[σ^2^(*F*_o_^2^) + (0.0289*P*)^2^ + 1.0903*P*] where *P* = (*F*_o_^2^ + 2*F*_c_^2^)/3
4198 reflections	(Δ/σ)_max_ = 0.001
199 parameters	Δρ_max_ = 0.60 e Å^−^^3^
0 restraints	Δρ_min_ = −0.58 e Å^−^^3^

### Special details

**Table:**

Geometry. All e.s.d.'s (except the e.s.d. in the dihedral angle between two l.s. planes) are estimated using the full covariance matrix. The cell e.s.d.'s are taken into account individually in the estimation of e.s.d.'s in distances, angles and torsion angles; correlations between e.s.d.'s in cell parameters are only used when they are defined by crystal symmetry. An approximate (isotropic) treatment of cell e.s.d.'s is used for estimating e.s.d.'s involving l.s. planes.
Refinement. Refinement of *F*^2^ against ALL reflections. The weighted *R*-factor *wR* and goodness of fit *S* are based on *F*^2^, conventional *R*-factors *R* are based on *F*, with *F* set to zero for negative *F*^2^. The threshold expression of *F*^2^ > σ(*F*^2^) is used only for calculating *R*-factors(gt) *etc*. and is not relevant to the choice of reflections for refinement. *R*-factors based on *F*^2^ are statistically about twice as large as those based on *F*, and *R*- factors based on ALL data will be even larger.

### Fractional atomic coordinates and isotropic or equivalent isotropic displacement parameters (Å^2^)

**Table:**

	*x*	*y*	*z*	*U*_iso_*/*U*_eq_	
Br1	−0.117304 (18)	0.402498 (14)	0.12147 (3)	0.03315 (8)	
N1	−0.15740 (18)	0.79184 (15)	0.2893 (4)	0.0560 (7)	
N2	0.27771 (15)	0.56650 (13)	0.4706 (3)	0.0366 (5)	
C1	−0.05428 (17)	0.50150 (13)	0.2166 (3)	0.0265 (4)	
C2	0.04561 (16)	0.49766 (13)	0.2815 (3)	0.0251 (4)	
H2	0.0823	0.4461	0.2787	0.030*	
C3	0.09102 (17)	0.57163 (13)	0.3513 (3)	0.0243 (4)	
C4	0.03878 (16)	0.64751 (13)	0.3566 (3)	0.0266 (4)	
H4	0.0703	0.6971	0.4059	0.032*	
C5	−0.06113 (17)	0.64919 (14)	0.2879 (3)	0.0288 (4)	
C6	−0.10855 (17)	0.57649 (14)	0.2169 (3)	0.0292 (4)	
H6	−0.1766	0.5784	0.1697	0.035*	
C7	−0.11580 (18)	0.72847 (15)	0.2884 (4)	0.0377 (6)	
C8	0.19580 (17)	0.56869 (13)	0.4179 (3)	0.0272 (4)	
Br1A	0.370700 (19)	0.099566 (15)	0.08217 (4)	0.03739 (8)	
N1A	0.37898 (18)	0.49595 (14)	0.0782 (3)	0.0460 (6)	
N2A	0.78226 (14)	0.24025 (12)	0.3862 (3)	0.0319 (4)	
C1A	0.44634 (16)	0.19863 (13)	0.1352 (3)	0.0264 (4)	
C2A	0.40402 (17)	0.27736 (14)	0.1025 (3)	0.0277 (4)	
H2A	0.3364	0.2823	0.0543	0.033*	
C3A	0.46205 (16)	0.34971 (13)	0.1413 (3)	0.0257 (4)	
C4A	0.56122 (16)	0.34354 (13)	0.2127 (3)	0.0252 (4)	
H4A	0.6006	0.3929	0.2381	0.030*	
C5A	0.60110 (15)	0.26273 (13)	0.2458 (3)	0.0233 (4)	
C6A	0.54470 (16)	0.18963 (12)	0.2074 (3)	0.0247 (4)	
H6A	0.5729	0.1350	0.2300	0.030*	
C7A	0.41674 (18)	0.43201 (15)	0.1068 (3)	0.0317 (5)	
C8A	0.70312 (17)	0.25181 (12)	0.3230 (3)	0.0262 (4)	

### Atomic displacement parameters (Å^2^)

**Table:**

	*U*^11^	*U*^22^	*U*^33^	*U*^12^	*U*^13^	*U*^23^
Br1	0.03797 (14)	0.02912 (13)	0.03247 (13)	−0.01325 (9)	0.00308 (10)	−0.00301 (8)
N1	0.0359 (12)	0.0372 (12)	0.095 (2)	0.0070 (10)	0.0032 (13)	0.0007 (13)
N2	0.0309 (10)	0.0297 (10)	0.0483 (13)	0.0062 (8)	−0.0035 (9)	−0.0032 (9)
C1	0.0320 (11)	0.0244 (10)	0.0234 (10)	−0.0081 (8)	0.0047 (8)	0.0003 (7)
C2	0.0310 (11)	0.0209 (9)	0.0236 (10)	−0.0016 (8)	0.0042 (8)	0.0012 (7)
C3	0.0262 (10)	0.0238 (9)	0.0229 (10)	−0.0008 (8)	0.0029 (8)	0.0012 (7)
C4	0.0270 (11)	0.0223 (10)	0.0304 (11)	−0.0021 (8)	0.0023 (9)	−0.0009 (8)
C5	0.0253 (11)	0.0262 (10)	0.0350 (12)	0.0005 (8)	0.0039 (9)	0.0013 (8)
C6	0.0226 (10)	0.0322 (11)	0.0328 (11)	−0.0035 (9)	0.0026 (9)	0.0022 (9)
C7	0.0263 (12)	0.0314 (12)	0.0554 (16)	0.0004 (10)	0.0024 (11)	0.0001 (11)
C8	0.0297 (11)	0.0200 (9)	0.0319 (11)	0.0014 (8)	0.0016 (9)	−0.0015 (8)
Br1A	0.03607 (14)	0.03308 (13)	0.04293 (15)	−0.01449 (9)	0.00186 (10)	−0.00027 (9)
N1A	0.0482 (14)	0.0387 (12)	0.0506 (14)	0.0124 (10)	−0.0004 (11)	0.0062 (10)
N2A	0.0297 (10)	0.0259 (9)	0.0394 (11)	0.0012 (8)	−0.0039 (9)	0.0011 (8)
C1A	0.0275 (11)	0.0265 (10)	0.0256 (10)	−0.0053 (8)	0.0053 (8)	−0.0007 (8)
C2A	0.0222 (10)	0.0366 (12)	0.0244 (10)	−0.0015 (9)	0.0022 (8)	0.0013 (8)
C3A	0.0280 (11)	0.0254 (10)	0.0237 (10)	0.0039 (8)	0.0018 (8)	0.0007 (8)
C4A	0.0289 (11)	0.0226 (10)	0.0240 (10)	0.0000 (8)	0.0005 (8)	−0.0009 (8)
C5A	0.0233 (10)	0.0253 (10)	0.0212 (10)	0.0006 (8)	0.0017 (8)	−0.0001 (7)
C6A	0.0268 (10)	0.0218 (9)	0.0262 (10)	−0.0004 (8)	0.0056 (8)	0.0004 (7)
C7A	0.0316 (12)	0.0330 (12)	0.0302 (11)	0.0032 (10)	0.0003 (9)	0.0018 (9)
C8A	0.0308 (11)	0.0194 (9)	0.0282 (11)	0.0002 (8)	0.0013 (9)	0.0004 (7)

### Geometric parameters (Å, º)

**Table:**

Br1—C1	1.888 (2)	Br1A—C1A	1.886 (2)
N1—C7	1.143 (3)	N1A—C7A	1.141 (3)
N2—C8	1.134 (3)	N2A—C8A	1.139 (3)
C1—C2	1.385 (3)	C1A—C2A	1.379 (3)
C1—C6	1.386 (3)	C1A—C6A	1.389 (3)
C2—C3	1.398 (3)	C2A—C3A	1.397 (3)
C2—H2	0.9500	C2A—H2A	0.9500
C3—C4	1.386 (3)	C3A—C4A	1.393 (3)
C3—C8	1.449 (3)	C3A—C7A	1.447 (3)
C4—C5	1.393 (3)	C4A—C5A	1.397 (3)
C4—H4	0.9500	C4A—H4A	0.9500
C5—C6	1.396 (3)	C5A—C6A	1.395 (3)
C5—C7	1.447 (3)	C5A—C8A	1.449 (3)
C6—H6	0.9500	C6A—H6A	0.9500
			
C2—C1—C6	121.65 (19)	C2A—C1A—C6A	121.60 (19)
C2—C1—Br1	119.13 (16)	C2A—C1A—Br1A	120.23 (16)
C6—C1—Br1	119.21 (17)	C6A—C1A—Br1A	118.18 (15)
C1—C2—C3	118.31 (19)	C1A—C2A—C3A	119.1 (2)
C1—C2—H2	120.8	C1A—C2A—H2A	120.5
C3—C2—H2	120.8	C3A—C2A—H2A	120.5
C4—C3—C2	121.7 (2)	C4A—C3A—C2A	121.22 (19)
C4—C3—C8	119.39 (19)	C4A—C3A—C7A	120.2 (2)
C2—C3—C8	118.86 (19)	C2A—C3A—C7A	118.6 (2)
C3—C4—C5	118.3 (2)	C3A—C4A—C5A	118.05 (19)
C3—C4—H4	120.8	C3A—C4A—H4A	121.0
C5—C4—H4	120.8	C5A—C4A—H4A	121.0
C4—C5—C6	121.3 (2)	C6A—C5A—C4A	121.71 (19)
C4—C5—C7	118.9 (2)	C6A—C5A—C8A	117.39 (18)
C6—C5—C7	119.8 (2)	C4A—C5A—C8A	120.90 (18)
C1—C6—C5	118.6 (2)	C1A—C6A—C5A	118.36 (19)
C1—C6—H6	120.7	C1A—C6A—H6A	120.8
C5—C6—H6	120.7	C5A—C6A—H6A	120.8
N1—C7—C5	178.8 (3)	N1A—C7A—C3A	178.4 (3)
N2—C8—C3	179.7 (3)	N2A—C8A—C5A	177.3 (2)
			
C6—C1—C2—C3	1.1 (3)	C6A—C1A—C2A—C3A	0.7 (3)
Br1—C1—C2—C3	179.95 (15)	Br1A—C1A—C2A—C3A	−179.35 (16)
C1—C2—C3—C4	0.0 (3)	C1A—C2A—C3A—C4A	−0.2 (3)
C1—C2—C3—C8	−179.33 (19)	C1A—C2A—C3A—C7A	−179.9 (2)
C2—C3—C4—C5	−0.8 (3)	C2A—C3A—C4A—C5A	−0.5 (3)
C8—C3—C4—C5	178.5 (2)	C7A—C3A—C4A—C5A	179.2 (2)
C3—C4—C5—C6	0.7 (3)	C3A—C4A—C5A—C6A	0.7 (3)
C3—C4—C5—C7	−178.5 (2)	C3A—C4A—C5A—C8A	−178.87 (19)
C2—C1—C6—C5	−1.3 (3)	C2A—C1A—C6A—C5A	−0.4 (3)
Br1—C1—C6—C5	179.87 (17)	Br1A—C1A—C6A—C5A	179.62 (15)
C4—C5—C6—C1	0.4 (3)	C4A—C5A—C6A—C1A	−0.3 (3)
C7—C5—C6—C1	179.5 (2)	C8A—C5A—C6A—C1A	179.29 (19)

### Hydrogen-bond geometry (Å, º)

**Table:**

*D*—H···*A*	*D*—H	H···*A*	*D*···*A*	*D*—H···*A*
C4*A*—H4*A*···N2^i^	0.95	2.69	3.388 (3)	130
C4—H4···N2*A*^i^	0.95	2.61	3.444 (3)	147
C6*A*—H6*A*···N1*A*^ii^	0.95	2.67	3.563 (3)	157
C2—H2···N1^iii^	0.95	2.69	3.624 (3)	168
C2*A*—H2*A*···N1^iii^	0.95	2.72	3.435 (3)	133

Symmetry codes: (i) −*x*+1, −*y*+1, −*z*+1; (ii) −*x*+1, *y*−1/2, −*z*+1/2; (iii) −*x*, *y*−1/2, −*z*+1/2.
